# Supplementary material for: Detection of locomotion deficit in a post-traumatic syringomyelia rat model using automated gait analysis technique
Source: PLoS One. 2021 Nov 11;16(11):e0252559. doi: 10.1371/journal.pone.0252559 (PMC8584658; doi:10.1371/journal.pone.0252559)
Supplement: S1 File — (DOCX) [file pone.0252559.s001.docx]

**Supporting Information**

**Detection of Locomotion Deficit in a Post-Traumatic Syringomyelia Rat Model Using Automated Gait Analysis Technique**

Dipak D. Pukale^1^, Mahmoud Farrag^2^, Nic D. Leipzig^1,2,*^

^1^Department of Chemical, Biomolecular, and Corrosion Engineering, University of Akron, Akron, OH, USA 44325

^2^Integrated Bioscience Program, University of Akron, Akron, OH, USA 44325

*Corresponding author: Nic D. Leipzig, Tel.: +1-330-972-6881; E-mail address: nl21@uakron.edu

**S1 Table. Dataset**

| **Duty Imbalance Forelimb** | | | | | |
| --- | --- | --- | --- | --- | --- |
| Groups/Weeks | 1 | 2 | 3 | 5 | 6 |
| Mean - Uninjured | 0.0517 | -0.0112 | 0.0953 | -0.0126 | 0.0412 |
| Mean - PTSM | 0.0020 | -0.0171 | -0.0263 | -0.0994 | -0.4423 |
| SD - Uninjured | 0.0360 | 0.0386 | 0.0308 | 0.0438 | 0.0174 |
| SD - PTSM | 0.0071 | 0.0187 | 0.0301 | 0.0340 | 0.0731 |
|  |  |  |  |  |  |
| **Duty Imbalance Hindlimb** | | | | | |
| Groups/Weeks | 1 | 2 | 3 | 5 | 6 |
| Mean - Uninjured | -0.0422 | -0.0313 | -0.0315 | -0.0773 | -0.0181 |
| Mean - PTSM | -0.0165 | -0.0217 | -0.0659 | -0.0658 | 0.1162 |
| SD - Uninjured | 0.0215 | 0.0171 | 0.0190 | 0.0196 | 0.0243 |
| SD - PTSM | 0.0134 | 0.0409 | 0.0130 | 0.0312 | 0.0680 |
|  |  |  |  |  |  |
| **Right Paw Placement Accuracy** | | | | | |
| Groups/Weeks | 1 | 2 | 3 | 5 | 6 |
| Mean - Uninjured | 1.1882 | 1.0273 | 0.8094 | 1.0571 | 0.7898 |
| Mean - PTSM | 1.2381 | 0.9748 | 1.4745 | 1.3977 | 2.1470 |
| SD - Uninjured | 0.5508 | 0.4358 | 0.2259 | 0.3139 | 0.4404 |
| SD - PTSM | 0.5755 | 0.7428 | 0.5820 | 0.2845 | 0.8043 |
|  |  |  |  |  |  |
| **Left Paw Placement Accuracy** | | | | | |
| Groups/Weeks | 1 | 2 | 3 | 5 | 6 |
| Mean - Uninjured | 1.0769 | 0.9415 | 0.9762 | 0.9610 | 1.0448 |
| Mean - PTSM | 1.3074 | 1.2331 | 1.2496 | 1.3997 | 1.3486 |
| SD - Uninjured | 0.4118 | 0.1522 | 0.1121 | 0.1418 | 0.2559 |
| SD - PTSM | 0.4927 | 0.2292 | 0.2914 | 0.2024 | 0.5260 |
|  |  |  |  |  |  |
| **Forelimb Step Contact Width** | | | | | |
| Groups/Weeks | 1 | 2 | 3 | 5 | 6 |
| Mean - Uninjured | 61.2058 | 57.0861 | 57.5622 | 64.6225 | 60.9177 |
| Mean - PTSM | 63.7648 | 53.7610 | 45.7974 | 43.1556 | 35.1703 |
| SD - Uninjured | 16.4395 | 13.9862 | 14.1037 | 12.5016 | 12.0564 |
| SD - PTSM | 7.9022 | 13.7171 | 10.9159 | 14.0622 | 14.7856 |
|  |  |  |  |  |  |
| **Hindlimb Step Contact Width** | | | | | |
| Groups/Weeks | 1 | 2 | 3 | 5 | 6 |
| Mean - Uninjured | 76.9998 | 80.5330 | 85.4406 | 82.5362 | 56.9640 |
| Mean - PTSM | 88.1975 | 71.5099 | 72.3360 | 79.7481 | 50.6033 |
| SD - Uninjured | 11.3200 | 14.7517 | 12.8566 | 8.9619 | 13.6774 |
| SD - PTSM | 14.1166 | 11.5870 | 18.8295 | 20.0421 | 20.7892 |
|  |  |  |  |  |  |
| **Forelimb Stride Length** | | | | | |
| Groups/Weeks | 1 | 2 | 3 | 5 | 6 |
| Mean - Uninjured | 984.6374 | 972.1677 | 1010.1742 | 969.3449 | 991.6046 |
| Mean - PTSM | 956.9147 | 952.8944 | 939.1307 | 915.8864 | 913.1958 |
| SD - Uninjured | 41.4575 | 78.4715 | 38.2771 | 59.1250 | 76.3411 |
| SD - PTSM | 28.8503 | 79.8968 | 66.5623 | 79.5859 | 54.6633 |
|  | |  |  |  |  |
| **Hindlimb Stride Length** | | | | | |
| Groups/Weeks | 1 | 2 | 3 | 5 | 6 |
| Mean - Uninjured | 882.4413 | 886.0821 | 882.4953 | 931.2352 | 924.6085 |
| Mean - PTSM | 865.4558 | 863.6762 | 878.2790 | 851.4219 | 782.9408 |
| SD - Uninjured | 46.1463 | 42.5093 | 68.1418 | 87.1642 | 58.0512 |
| SD - PTSM | 43.4481 | 82.7339 | 67.1694 | 65.2097 | 97.4442 |
